# 2389. Surveillance evaluation of COVID-19 pharmacovigilance—Karakalpakstan, Uzbekistan, 2022

**DOI:** 10.1093/ofid/ofad500.2009

**Published:** 2023-11-27

**Authors:** Yunis Tursinov, Botirjon Kurbanov, Alfiya Denebayeva, Roberta Horth, Dilyara Nabirova

**Affiliations:** Central Asia Field Epidemiology Training Program, Нукус, Qoraqalpoghiston, Uzbekistan; Sanitary-Epidemiological Tranquility and Public Health Committee Tashkent, Uzbekistan, Tashkent, Toshkent, Uzbekistan; Almaty City Center for Prevention and Control of AIDS, HIV Center, Kazakhstan,, Almaty, Almaty, Kazakhstan; US Centers for Disease Control and Prevention, Dulles, Virginia; CDC Central Asia office, Almaty, Almaty, Kazakhstan

## Abstract

**Background:**

COVID-19 vaccines are safe and effective. Rapid rollout of vaccines reduced COVID-19 morbidity and mortality globally. Pharmacovigilance systems have ensured safety of these vaccines. Well-functioning vaccine safety surveillance builds public confidence in vaccine programs, but these systems may be lacking in limited-resource settings. To identify gaps in pharmacovigilance surveillance, we conducted an evaluation in Khojaly District, Uzbekistan, with population of 120 thousand people.

**Methods:**

We reviewed regulatory documents related to COVID-19 vaccination and registration of side effects after immunization. We also conducted a survey in April 2022 of 30 healthcare providers in 5 of 10 polyclinics in Khojaly districts whose responsibilities included vaccination of the population. The survey asked about their knowledge and practices related to adverse events following immunization (AEFI). AEFI was defined as any health condition that occurs after immunization and does not necessarily have a causal relationship with vaccination.

**Results:**

From April 2021 to March 2022, 78,453 doses of COVID-19 vaccines were given in Khojaly district. Of these, 70% of doses were from ZF-UZ-VAC-2001 (Zifivax) vaccine doses, an adjuvanted protein produced in Uzbekistan (Table 1), 9% Pfizer–BioNTech vaccine doses and 7% Moderna vaccine doses. All AEFIs are mandatorily reported by providers (Figure 1). Of 2,464 reported AEFI, majority (75%) were procedural errors, 1% were mild allergic reactions, and only 2 cases of anaphylactic shock. Among healthcare providers surveyed (Table 2), 33% did not know where to report the AEFI and 10% reported large patient load as a barrier to reporting. Half (50%) of providers had encountered a case of AEFI following COVID-19 vaccination, 10% encountered a severe AEFI, and 43% submitted an AEFI report. In record review, we identified cases of AEFI that had only been registered in patient medical charts, and not reported into the AEFI surveillance system.

Diagram of AEFI surveillance for COVID-19 vaccines in Uzbekistan

**Figure:**
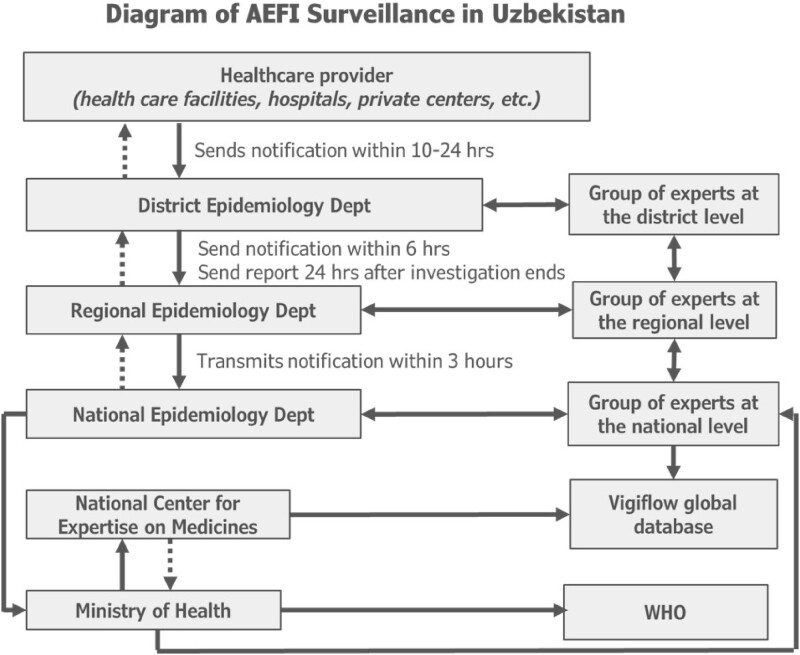

COVID-19 vaccine doses and reported AEFI, Khojaly district, Uzbekistan, 2021-22

**Figure:**
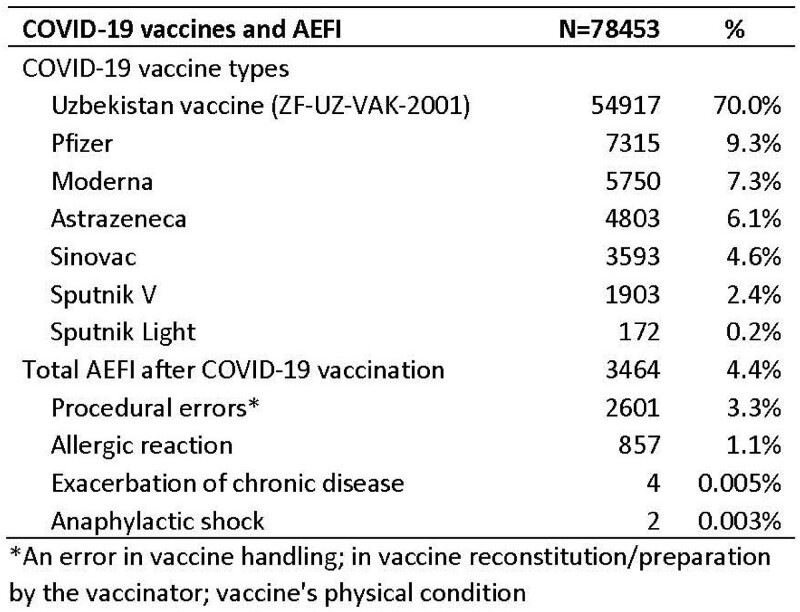

Knowledge of AEFI and AEFI reporting among COVID-19 vaccine providers, Khojaly district, Uzbekistan, 2021-22

**Figure:**
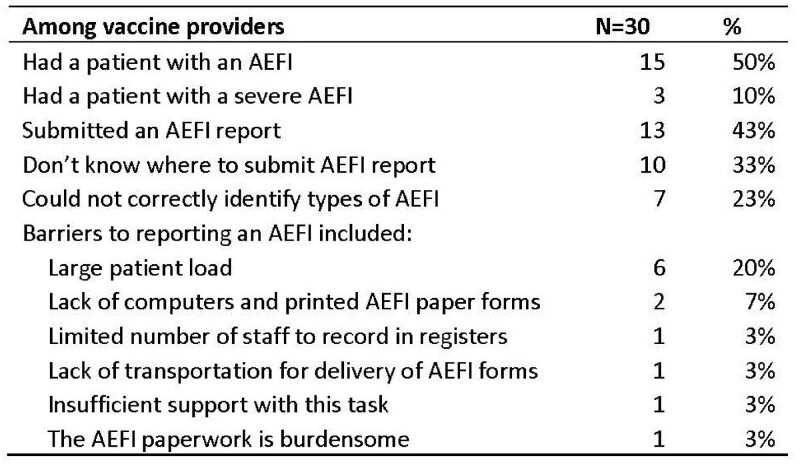

**Conclusion:**

Important considerations were identified in COVID-19 pharmacovigilance surveillance. The system can be strengthened through increased training of healthcare providers in standard operating procedures for identifying, reporting and investigation AEFI cases associated with COVID-19 vaccines.

**Disclosures:**

**All Authors**: No reported disclosures

